# Benign monomelic amyotrophy of lower limb in a cohort of chinese patients

**DOI:** 10.1002/brb3.2073

**Published:** 2021-03-02

**Authors:** Lulu Wang, Han Wen, Shuyun Chen, Huan Wang, Yilei Zheng, Ran Chen, Jingjing Li, Kaiyan Jiang, Haijie Xiang, Min Zhu, Meihong Zhou, Sheng Yao, Daojun Hong

**Affiliations:** ^1^ Department of Neurology The First Affiliated Hospital of Nanchang University Nanchang China; ^2^ Department of Neurology The Sixth Medical Center of General PLA Hospital Beijing China; ^3^ Department of Neurology Peking University People Hospital Beijing China

**Keywords:** amyotrophic lateral sclerosis, anterior horn cell disorder, benign monomelic amyotrophy of lower limb, muscle MRI, neurogenic pattern

## Abstract

**Background:**

Benign monomelic amyotrophy of lower limb (BMALL) is a neurogenic syndrome representing an unclear field. Further studies might be helpful to elucidate uncertainties regarding causation, outcome, and the risk of progression to amyotrophic lateral sclerosis (ALS).

**Methods:**

According to the inclusion and exclusion criteria, 37 patients with BMALL were retrospectively collected in three neuromuscular centers from January 2012 to October 2018. The detailed medical data were summarized. Multiple laboratory tests were examined. Routine electrophysiological examinations, muscle MRI of lower limbs, and muscle biopsy were conducted.

**Results:**

The cohort included 24 male and 13 female cases with median age of onset 47 years. Muscle MRI revealed that the distribution of involved muscles matched with the extent of fat infiltration, so the pattern muscle atrophy can be divided into the following four types: six patients with thigh atrophy (type I), 14 patients with leg atrophy (type II); 10 patients with disproportionate atrophy in both thigh and leg (type III); and seven patients with well‐proportionate atrophy in both thigh and leg (type IV). Electrophysiological findings showed neurogenic pattern, spontaneous activity, and abnormal H reflex, which suggested a disorder of spinal anterior horn cell in the patients with types I‐III. However, no electrophysiological abnormalities were found in the patients with type IV. Muscle pathology varied from almost normal pattern to advanced neurogenic pattern in nine biopsied patients. Follow‐up showed that two patients with type II developed to ALS four years later, and all patients with type IV were in stable condition without any complaints.

**Conclusion:**

Muscle MRI was useful to exactly localize the distribution of involved muscles in BMALL patients. The distribution of atrophic muscles can be roughly divided into four types based on the MRI features. The classification of distributing types might be as an indicator for the prognosis of BMALL.

## Graphical abstract

Benign monomelic amyotrophy of lower limb (BMALL) is a neurogenic syndrome representing an unclear field. This study might be helpful to elucidate uncertainties regarding causation, outcome, and the risk of progression to amyotrophic lateral sclerosis (ALS).

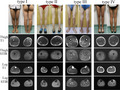

## 1 INTRODUCTION

Benign monomelic amyotrophy of lower limb (BMALL) is a rare neurological syndrome (Felice et al., 2003; Gourie‐Devi, 2007). It is clinically characterized by insidious‐onset wasting restricted to a single lower limb with a slow progressive or arrested course, although a few deteriorating cases have occasionally been described (De Carvalho & Swash, 2007; Kay et al., 1994). The cause of BMALL and its relation to other anterior horn cell disorders, such as amyotrophic lateral sclerosis (ALS), are poorly understood (Guennoc et al., 2013). The possible pathogenesis seems to be associated with genetic factors (Fetoni et al., 2004), immunological antibodies (Khandelwal et al., 2006; Weiss, 2005), viral infection (McMillan et al., 2009; Vibha, 2015), and focal ischemia (Bella et al., 1992).

The majority of BMALL cases were found in male Indian (Gourie‐Devi et al., 1984; Nalini et al., 2014; Prabhakar et al., 1981; Saha et al., 1997; Virmani & Mohan, 1985), although some cases were also reported in East Asia (Hamano et al., 1999; Kim et al., 1994) and Western countries (Freitas & Nascimento, 2000; Visser et al., 1988; Muzio et al., 1994; Dimachkie et al., 2000; Guglielmo et al., 1996; Moglia et al., 2011; Münchau & Rosenkranz, 2000; Riggs et al., 1984; Uncini et al., 1992). Only a few cases were reported in Mainland China up to now (Hui et al., 2018), although China has the largest population in the world. BMALL represents an unclear field among neurogenic syndromes; further studies might be helpful to elucidate uncertainties regarding causation, outcome, and the risk of progression to ALS. In this study, we aimed to describe the clinical characteristics, electrophysiological changes, MRI features, and histopathological patterns in a cohort of 37 Chinese patients with BMALL.

## 2 MATERIALS AND METHODS

### 2.1 Subjects

All patients with monomelic amyotrophy of lower limbs were collected from three academic neuromuscular clinics (the First Affiliated Hospital of Nanchang University, the Sixth Medical Center of General PLA Hospital, and Peking University People's Hospital) between January 2012 and October 2018. The last follow‐up was in October 2020. The following laboratory tests were conducted in all patients: erythrocyte sedimentation rate, hemoglobin, blood sugar, thyroid function hormone, parathyroid hormone, and serum immunoelectrophoresis with immunofixation. Serum IgM of antipoliovirus was detected in 12 acute‐onset patient. Serum IgM of anti‐GM1 antibody was examined in 11 patients. Cerebrospinal fluid (CSF) was examined in 10 patients. Electromyogram (EMG) and nerve conduction study (NCS) were conducted in all patients. Lumber MRI was performed in all patients.

The inclusion criteria were (a) clinical evidence of wasting with or without weakness restricted to a single lower limb; (b) stationary course or initial progression followed by a stationary course; and (c) at least 24 months of a follow‐up period. The exclusion criteria were (a) deletion in the survival motor neuron 1 (*SMN1*) gene or an expansion of CAG‐repeats (>40) in the androgen receptor (*AR*) gene; (b) history of diseases that may mimic lower motor neuron disease (LMND) including acute poliomyelitis, spinal radiculopathy, thyrotoxicosis, and hyperparathyroidism; (c) history of diabetes mellitus; (d) clinical signs of upper motor neuron involvement including pseudobulbar symptoms, brisk jaw jerk, hyperreflexia, and extensor plantar response; (e) objective sensory signs on neurological examination (NCS evidence of denervation in the opposite limb was not a reason for exclusion); (f) tracheostomy or intermittent ventilatory assistance; (g) structural lesions (tumors, intervertebral disk herniation, vascular lesions, and syringomyelia) on spinal MRI; and (h) motor conduction block on standardized NCS.

### 2.2 Ethical statement

All patients’ tissue samples were obtained after a written consent signed by each individual in compliance with the bioethics laws of China and the Declaration of Helsinki. The research was approved by ethics committee of the First Affiliated Hospital of Nanchang University.

### 2.3 Muscle MRI

Axial planes of the thigh and leg muscles were imaged in all patients using 3.0‐T MR scanners. Conventional T1‐weighted image (T1WI) sequences were obtained to observe fatty infiltration with the following parameters: repetition time (TR) = 500ms, echo time (TE) = 8 ms, and matrix: 512 × 512. The extent of fatty infiltration was evaluated according to the modified Mercuri scale (0–5 scores) (Mercuri et al., 2002). A fatty score was adopted as follows: normal (score 0); punctate hyperintense (score 1); fatty streaks detected below 30% muscle volume (score 2); hyperintense detected among 30% to 60% muscle volume (score 3); hyperintense detected above 60% muscle volume (score 4); and hyperintense in the whole muscle (score 5). The short time inversion recovery (STIR) sequences were obtained to evaluate muscle edema with the following parameters: TR = 6100ms, TE = 70ms, inversion time = 180ms, and matrix: 512 × 512. The slice thickness was 5mm with a slice gap of 1mm, and the field of view was 36 × 48cm.

### 2.4 Muscle biopsy

Muscle biopsies were conducted in nine patients. The tissues were frozen and then cut into 8µm sections. These sections were stained according to standard histological and enzyme histochemical procedures with hematoxylin and eosin (H&E), modified Gomori trichrome, periodic acidic Schiff, oil red O, adenosine triphosphatase (ATPase), nicotinamide adenine dinucleotide tetrazolium reductase, succinate dehydrogenase, cytochrome *c* oxidase, and nonspecific esterase. Immunohistochemical stain was applied with myosin heavy chain 7 antibody (MYH7, Abcam) to indicate the type 1 fibers.

### 2.5 Statistical analysis

Data were analyzed using SPSS version 22.0 (SPSS Inc., Chicago, IL, USA). The normality of variables distribution was evaluated using the Kolmogorov–Smirnov test. Dichotomous variables were expressed as percentages and absolute frequencies. Continuous data were expressed as medians (quartile interval Q1, Q3). Comparisons of categorical variables between groups were conducted by the chi‐square test or Fisher's exact test, and Bonferroni test, as appropriate. The Kruskal–Wallis H test was used to compare continuous variables among groups. To adjust for the confounders in this retrospective study, multiple linear regression model was established to identify the difference. Independent *t* test was conducted to assess the difference in electrophysiological values between different nerves. Differences were considered statistically significant if *p* <.05.

## 3 RESULTS

### 3.1 Patient enrollment

A total of 49 patients were initially recruited based on the inclusion criteria, but 12 of them were excluded due to the following reasons: Five patients had signs of upper motor neuron involvement; three patients had a history of poliomyelitis; two patients had diabetic amyotrophy; one patient had definite sensory signs; and one patient had lumber myelopathy. Consequently, 37 patients with monomelic amyotrophy of lower limbs were included in the observational cohort. Among 37 patients, no variants were detected in the *AR* or *SMN1* gene, and no abnormalities of lumber spinal cord or cauda equina root were found by lumbosacral MRI. No positive IgM antibodies of anti‐GM1 or antipoliovirus were identified in available patients. No elevated CSF protein or cell count was found in the examined patients.

### 3.2 Clinical features

The cohort included 24 male and 13 female patients without family history. The median age of first hospital visiting was 51 (44, 56) years. The median age of onset was 47 (38, 53) years. The occupation of the patients included labor worker (17 cases), student (9 cases), athlete (3 cases), staff member (3 cases), and unemployment (5 cases). Nine patients had no symptoms until their limb wasting was occasionally noticed by themselves or others, so the definite age of onset was difficult to determine in some patients. All patients exhibited muscle atrophy of a single lower limb, but 11 patients simultaneously had complaint of muscle weakness at the first visiting. Eighteen patients had concomitant symptoms including cold paresis in seven patients, muscle fasciculation in four patients, subjective numbness in four patients, muscle soreness in three patients, local skin pigmentation in two patients, and myalgia in one patient. The main clinical characteristics of patients are summarized in Table 1.

**TABLE 1:** Clinical features of patients with benign monomelic amyotrophy of lower limb

Case	Gender/age	Duration	Occupation	Wasting distribution	Onset manifestation	Concomitant symptoms	CK	Follow‐up time (mo)	Prognosis
**Type I**									
1	M/46	2 months	Cooker	L thigh	Wasting, Weakness	None	86	92	P‐ST
2	F/50	8 days	Housewife	L thigh	Wasting	CP	123	91	P‐ST
3	F/20	15 days	Student	L thigh	Wasting	CP	155	79	ST
4	M/18	0 day	Student	R thigh	Wasting	None	157	57	ST
5	F/44	0 day	Driver	L thigh	Wasting	None	189	47	P‐ST
6	M/16	1 month	Student	L thigh	Wasting, weakness	Myalgia	210	47	P‐ST
**Type II**									
7	M/47	3 months	Orchard man	R leg	Wasting, weakness	Numbness	234	94	ALS
8	M/44	2 days	Waiter	R leg	Wasting	None	88	91	P‐ST
9	M/57	5 years	Peasant	R leg	Wasting	None	74	90	ST
10	M/55	10 years	Woodcutter	L leg	Wasting	Numbness	83	39 lost	ST
11	M/40	2 years	Fisher	R leg	Wasting	None	151	89	P‐ST
12	F/46	13 years	Unemployed	R leg	Wasting, weakness	CP	96	86	ST
13	F/42	3 months	Builder	R leg	Wasting, weakness	CP, MF	340	84	ALS
14	M/50	4 months	Peasant	L leg	Wasting	SP	176	74	P‐ST
15	M/59	11 months	Builder	R leg	Wasting	None	294	68	P‐ST
16	F/53	4 days	Soldier	R leg	Wasting	CP	51	61	P‐ST
17	F/65	5 days	Peasant	R leg	Wasting	None	189	52	ST
18	M/58	20 years	Worker	R leg	Wasting	CP	304	43	ST
19	M/52	10 years	Athlete	L leg	Wasting	None	201	38	P‐ST
20	M/37	6 years	Unemployed	R leg	Wasting, weakness	MF	750	38	P‐ST
**Type III**									
21	F/56	6 years	Peasant	R leg–thigh	Wasting, weakness	CP, MF	145	56 lost	P‐ST
22	M/34	10 months	Salesperson	R leg–thigh	Wasting, weakness	SP	60	89	P‐ST
23	M/61	0 day	Peasant	R leg–thigh	Wasting	Numbness	61	87	P‐ST
24	M/53	6 months	Peasant	L leg–thigh	Wasting	None	65	84	P‐ST
25	M/49	27 years	Professor	R leg–thigh	Wasting	None	60	73	ST
26	F/48	15 years	Worker	L leg–thigh	Wasting, weakness	Soreness	86	62	ST
27	F/54	2 months	Athlete	R leg–thigh	Wasting, weakness	Sore**n**ess, MF	132	56	P‐ST
28	M/53	1 day	Driver	R leg–thigh	Wasting	None	60	52	P‐ST
29	M/68	4 months	Unemployed	L leg–thigh	Wasting, weakness	Numbness	202	43	P‐ST
30	M/28	5 years	Athlete	R leg–thigh	Wasting	None	114	35	P‐ST
**Type IV**									
31	F/14	2 years	Student	R leg–thigh	Wasting	None	56	61	ST
32	F/13	10 years	Student	R leg–thigh	Wasting	None	76	44	ST
33	F/15	1 year	Student	R leg–thigh	Wasting	None	55	42	ST
34	M/13	1 year	Student	R leg–thigh	Wasting	None	203	32	ST
35	M/51	50 years	Unemployed	L leg–thigh	Wasting	Soreness	172	30	ST
36	M/26	20 year	Student	R leg–thigh	Wasting	None	163	26	ST
37	M/18	12 years	Student	R leg–thigh	Wasting	None	95	24	ST

Note

Duration: the period is from symptom presentation to first consultation. Follow‐up time is from first consultation to March 2020.

Abbreviation: ALS, amyotrophic lateral sclerosis; CK, creatine kinase; CP, cold paresis; SP, skin pigmentation; MF, muscle fasciculation; mo, month; M, male; F, female; R, right; L, left.

The values of erythrocyte sedimentation rate, hemoglobin, blood sugar, thyroid function hormone, parathyroid hormone, and serum immunoglobulins were within normal limits. The level of CK was elevated in five patients with 234, 340, 294, 304, and 750 IU/L, respectively, and others had a range from 51 to 210 IU/L (normal 30–210 IU/L). Serum IgM of antipoliovirus was negative in 12 examined patients. Serum IgM of anti‐GM1 antibody was negative in 11 examined patients. CSF analysis of 10 patients showed that the level of glucose and chloride all were normal; the white blood cell counts with normal range from 0 to 8 x 10^6^/L in eight patients, the other two patients were 9 and 12 x 10^6^/L, respectively; the level of protein with normal range from 150 to 450 mg/L in nine patients, the other one was 520 mg/L.

**FIGURE 1:**
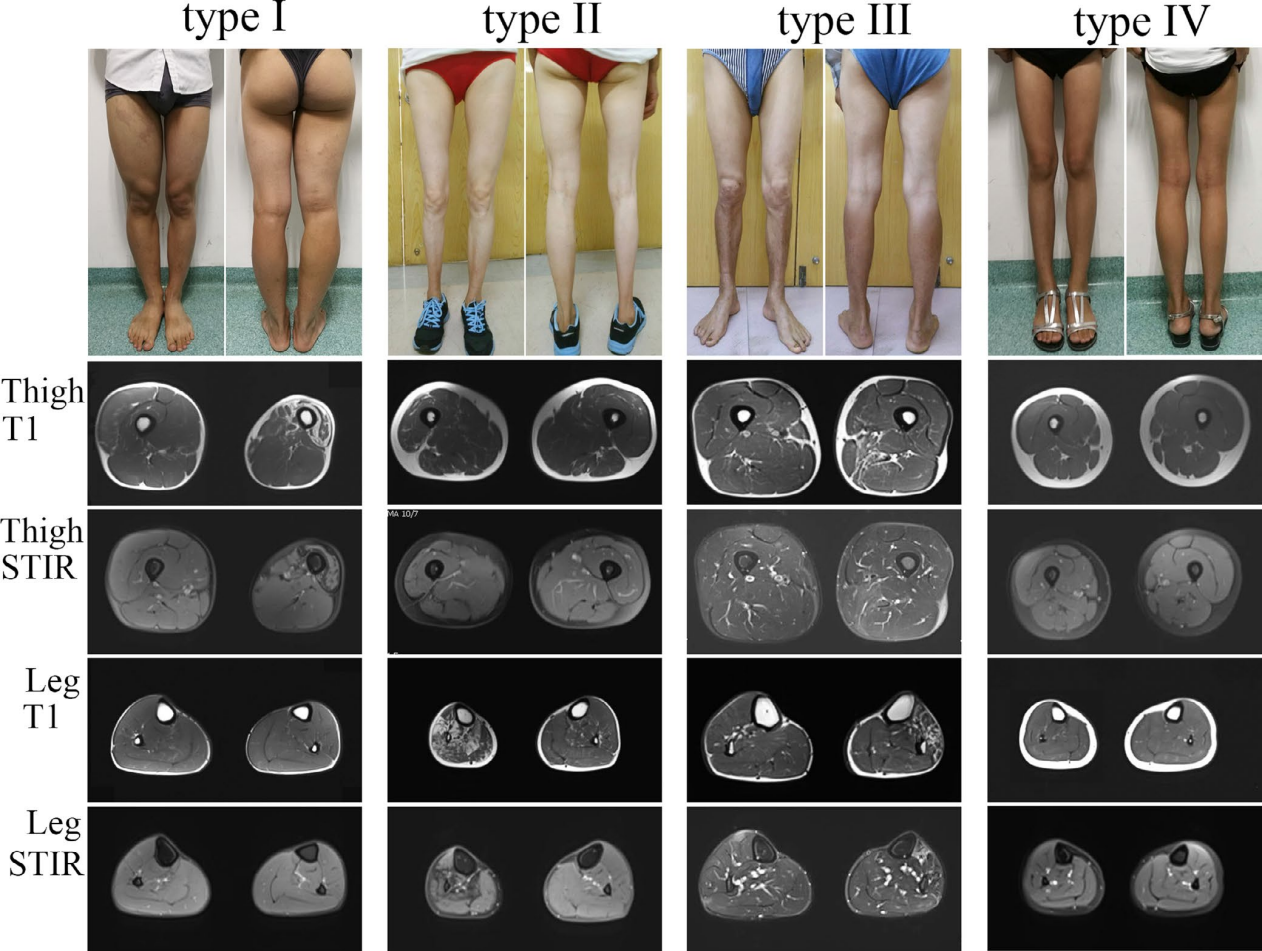
The distribution of involved muscles can be divided into four types. Type I was restricted to thigh atrophy represented by case 1 with fat infiltration in quadriceps femoris. Type II was restricted to leg atrophy represented by case 10 with diffused fat infiltration in the leg. Type III was disproportionate atrophy in both thigh and leg represented by case 24 with a combination fat infiltration of thigh posterior muscle group and leg anterior muscle group. Type IV was well‐proportionate atrophy in both thigh and leg represented by case 31 without fat infiltration. The patients gave their written informed consent for the publication

Based on the distribution of involved muscles from a clinical view, we identified four types of muscle atrophy (Figure 1): restricted to thigh atrophy (type I); restricted to leg atrophy (type II); disproportionate atrophy in both thigh and leg (type III); and well‐proportionate atrophy in both thigh and leg (type IV). The type I group included 6 patients (3 males and 3 females). The median age of onset was 44 years. The wasting distribution showed anterior thigh atrophy in four patients and posterior thigh atrophy in two patients. The type II group included 14 patients (10 males and 4 females). The median age of onset was 45 years. The wasting distribution showed whole leg atrophy in seven patient, posterior leg atrophy in six patients, and anterior leg atrophy in one patient. The type III group included 10 patients (7 males and 3 females). The median age of onset was 33 years. The wasting distribution showed posterior thigh and posterior leg atrophy in four patients, posterior thigh and anterior leg atrophy in four patients, and anterior thigh and posterior leg atrophy in two patients. The type IV group included seven patients (4 males and 3 females). The median age of onset was 12 years. The wasting distribution showed a well‐proportionate atrophy of the whole lower limb in comparison with contralateral lower limb.

The patients with type IV had a younger age of onset with comparison to those of the other three patterns (Table 2). Linear regression model was configured to further evaluate the confounders that may have introduced bias into the retrospective study. After adjustment for sex, duration of illness, occupation, muscle weakness, and concomitant symptoms, the onset age of patients with type IV still was younger than the others (odds ratio: 2.316, 95% confidence interval: 1.102–11.371).

**TABLE 2:** The comparison of clinical variables between the different patterns

Variables	Type I (*n* = 6)	Type II (*n* = 14)	Type III (*n* = 10)	Type IV (*n* = 7)	*P* value
Male	3 (50.0%)	10 (71.4%)	7 (70.0%)	4 (57.1%)	.352
Age at onset (years)	44 (20, 50)	45 (38, 47)	33 (33, 50)	12 (6, 14)	.000
Duration of illness (months)	1 (0.5, 2)	11 (11, 240)	60 (2, 60)	360 (240, 600)	.000
Labor occupation	2 (40.0%)	12 (85.7%)	7 (70.0%)	0 (0%)	.000
Muscle weakness	2 (40.0%)	4 (28.6%)	5 (50.0%)	0 (0%)	.000
Concomitant symptoms	3 (60.0%)	8 (57.1%)	6 (60.0%)	1 (14.3%)	.036
CK (IU/L)	157 (155, 189)	96 (83, 151)	61 (60, 86)	163 (95, 172)	.903
Follow‐up time (months)	79 (57, 91)	81 (64, 90)	73 (56, 87)	42 (30, 44)	.068
Prognosis (ALS number)	0 (0%)	2 (14.3%)	0 (0%)	0 (0%)	.052

Abbreviation: ALS, amyotrophic lateral sclerosis; CK, creatine kinase.

### 3.3 Electrophysiological changes

In the patients with type I wasting, the median motor nerve conduction velocity (NCV) of peroneal and tibial nerves in the wasting limbs were 45.2 (38.3, 47.3) m/s and 44.8 (39.1, 48.6) m/s, respectively. The median compound motor action potential (CMAP) of peroneal and tibial nerves in the wasting limbs were 3.9 (2.8, 6.3) mv and 11.6 (8.1, 15.6) mv, respectively. The CMAP of femoral nerve mildly decreased to 4.5 (3.6, 6.8) in four patients who showed wasting of quadriceps muscles. The sensory NCV and sensory nerve action potential (SNAP) were normal. The H reflex was not evoked in two patients, and the latency was prolonged in the other four patients.

In the patients with type II wasting, the motor NCV of peroneal and tibial nerves in the wasting limbs was 40.3 (37.2, 45.8) and 41.7 (38.1, 48.3) m/s, respectively. The mean CMAP of peroneal and tibial nerves in the wasting limbs was 3.7 (2.5, 6.2) mv and 6.4 (4.1, 10.7) mv, respectively. The sensory NCV and SNAP were normal. The H reflex was not evoked in four patients, the latency was prolonged in the seven patients, and the latency was normal in three patients.

In the patients with type III wasting, the motor NCV of peroneal and tibial nerves in the wasting limbs was 41.3 (38.5, 46.2) and 43.2 (38.6, 47.4) m/s, respectively. The mean CMAP of peroneal and tibial nerves in the wasting limbs was 4.8 (3.8, 6.3) mv and 6.5 (4.7, 10.2) mv, respectively. The sensory NCV and SNAP were normal. The H reflex was not evoked in six patients, the latency was prolonged in the 3 patients, and the latency was normal in one patient.

In the patients with type IV wasting, the motor and sensory nerve conduction studies of the wasting limbs were normal. The H reflexes were normal in examined patients.

The results of needle EMG are listed in supplemental Table S1 in detail. Overall, most clinically involved muscles of lower limbs showed chronic reinnervative changes in patients with type I, type II, and type III, while the muscles of upper limbs were intact. The EMG revealed a neurogenic pattern of quadriceps muscles in 20 of 30 examined patient (66.7%); tibialis anterior muscles in 26 of 30 examined patients (86.7%); and gastrocnemius muscles in 21 of 30 examined patients (70.0%). However, the amplitude and duration of motor action potentials in quadriceps, tibialis anterior, and gastrocnemius muscles displayed a normal pattern in seven patients with type IV wasting. The spontaneous activities such as fibrillations, positive sharp wave, and fasciculation potentials were relatively rare and only identified in seven patients, while two patients with ALS progression showed active denervation.

### 3.4 Muscle MRI features

Muscle MRI revealed that the distribution of involved muscles exactly matched with the extent of fat infiltration. In patients with type I, foue patients had fat infiltration mainly in quadriceps femoris with scores ranging 2–4 (Figure 1, type I), and 2 patients had fat infiltration mainly in hamstring muscle group with score of 2 and 4, respectively. In patients with type II, six patients showed a diffused fat infiltration in the leg with scores ranging 2–5 (Figure 1, type II), sven patients showed fat infiltration in the posterior leg muscles with scores ranging 2–5, and one patient showed fat infiltration in the anterior leg muscles with score of 3. In patients with type III, four patients displayed a combination of fat infiltration in the posterior thigh with scores ranging 2–4 and anterior leg muscles with scores ranging 2–5 (Figure 1, type III), four patients displayed a combination of fat infiltration in the posterior thigh with scores ranging 2–4 and posterior leg muscles with scores ranging 2–4, and two patients displayed a combination of fat infiltration in the anterior thigh with scores of 2 and 5, and posterior leg muscles with scores of 2 and 5. In patients with type IV, all seven patients showed a well‐proportionate atrophy of both thigh and leg without fat infiltration with score 0 (Figure 1, type IV). The number of involved muscles with fat infiltration was 19 in gastrocnemius and soleus, 11 in tibialis anterior, 7 in semitendinosus and semimembranosus, 6 in quadriceps femoris, and 3 in adductor magnus. Obvious muscle or fascia edema was not observed in most patients. The detailed fat infiltration scores of individual muscle are listed in the supplemental Table S2 and Table S3.

### 3.5 Muscle pathological changes

**FIGURE 2:**
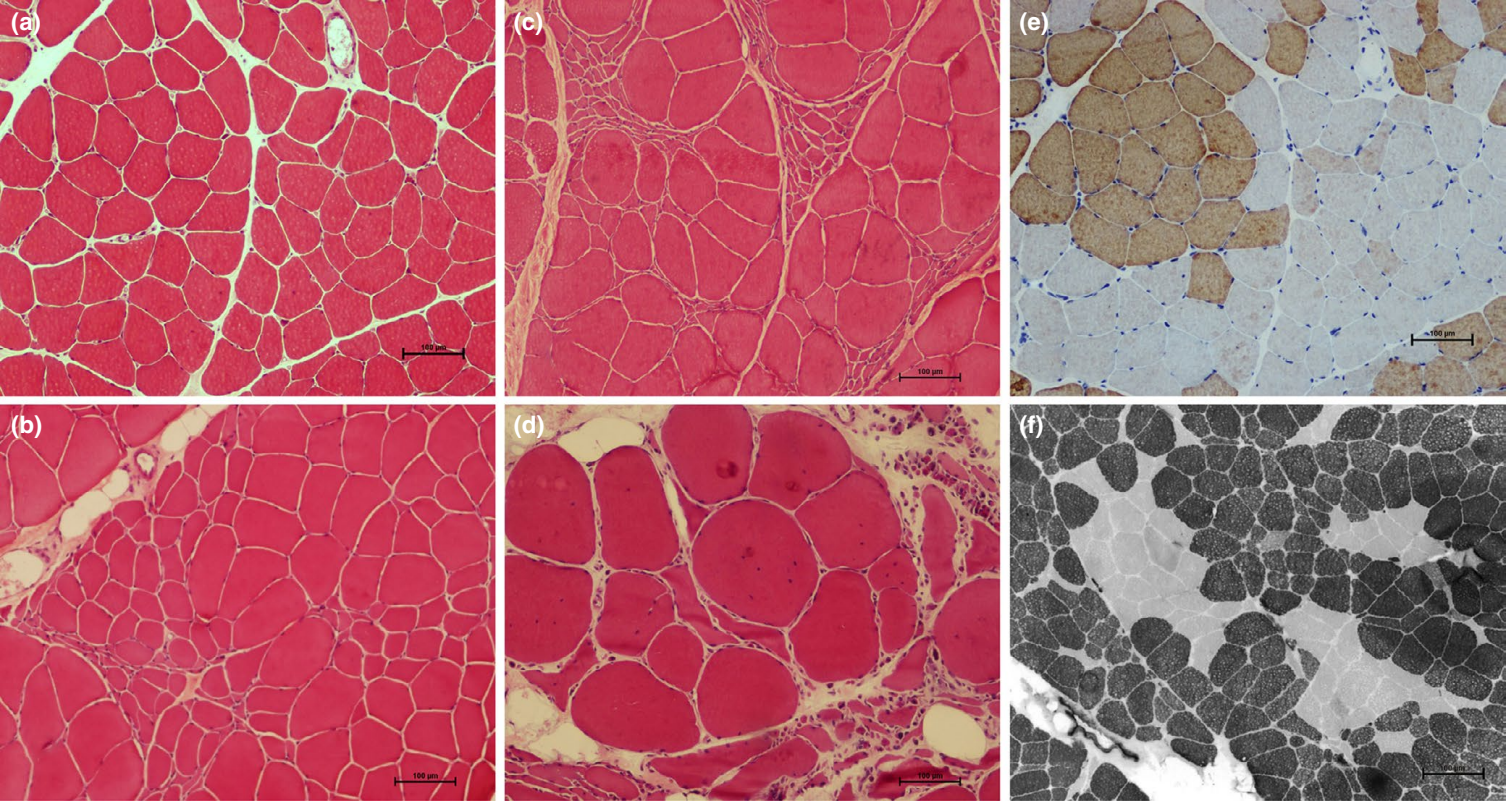
Muscle biopsy features in the BMALL patients. HE staining showed a normal structure (A), mild neurogenic atrophy characterized by groups of relatively small atrophy in some foci (B), typical neurogenic atrophy characterized by fish‐bank‐like angular atrophy of the whole muscle fascicle (C), and an advanced neurogenic pattern featured by groups of severely atrophic fibers with secondary myopathic changes (D). MHY7 immunostaining indicated grouping of type 1 fiber (E), and ATPase staining at PH 10.6 showed grouping of type 2 fiber (F)

The muscle pathological changes were variable in the nine patients underwent muscle biopsy. Two patients displayed almost normal pattern except for a few angular atrophy fibers (Figure 2a). One patient showed a mild neurogenic muscle atrophy characterized by groups of relatively small atrophy in some foci (Figure 2b). Three patients exhibited a typical neurogenic muscle atrophy characterized by a large group of angular atrophy in a whole muscle fascicle (Figure 2c). Three patients appeared an advanced neurogenic muscle pattern featured by groups of severely atrophic fibers with pyknotic nuclear clumps and chains, muscle hypertrophy, proliferation of connective tissue, internal migration of nuclei, and occasionally degenerative fibers (Figure 2d). The neurogenic groupings were further confirmed by MHY7 immunostaining for type 1 fiber (Figure 2e) and ATPase staining at PH 10.6 for type 2 fiber (Figure 2f).

### 3.6 Follow‐up

All patients had follow‐up time of at least two years. The median follow‐up time was 74 (52, 89) months. Twenty‐eight of 37 patients had more than 5 years of follow‐up plus disease duration. Sixteen patients with disease duration more than one year exhibited stable symptoms, and the other 21 patients with disease duration less than one year showed a relatively stable symptom with good physical ability. In the type I group, four patients showed a mild progression for about one year and then got stable; the other two patients were stable during the follow‐up. In the type II group, seven patients experienced a progression and then stable; five patients were stable during the follow‐up; but two patients gradually developed into ALS at the 48th month and 66th month, respectively. In the type III group, eight patients had a progression in two years, and then got stable; two patients were stable during the follow‐up. In the type IV group, all patients were stable without any complaints for at least three years. Patients with type II showed a tendency toward ALS, but the statistical variances were not significant with a borderline value (Table 2).

## 4 DISCUSSION

Unilateral atrophy of a lower limb could be noticed by the patients because of wasting with or without weakness on walking, but in over a half of the patients it was incidentally observed by their family members, friends, or physicians during consultation for unrelated illness (Gourie‐Devi, 2007; Nalini et al., 2014). In our study, only 11 patients initially complained of mild weakness of unilateral lower limb, and the others displayed insidious wasting of lower limb. Consequently, the precise age of onset and illness duration may not be very accurate. The age of onset of our patients was similar to that of European (Carvalho & Swash, 2007) or Japanese population (Hamano et al., 1999), but elder than that of Indian (Nalini et al., 2014) or Korean population (Kim et al., 1994). Remarkable gender preference with a male/female ratio 2:1 to 40:1 had been reported in BMALL patients from different population groups [12, 24]. In our case series, the male/female ratio was 1.7:1, which was similar to that of Korean population (Kim et al., 1994), and indicating that female patients might be relatively common in Eastern Asia (Table 3). The patients with type IV had a younger age of onset and a relatively equivalent sex ratio with comparison to those of other three types, which suggested that the condition of type IV might be a distinct subgroup.

**TABLE 3:** The summary of clinical data for BMALL patients reported in English literatures

Literature	Geography	Case number	Gender	Age	Illness duration	Main atrophy distribution	Other symptom	Prognosis
Prabhakar et al. (1981)	India	40	*M*(39), *F*(1)	24.5y	6w−14y	Leg(9), thigh(5), leg–thigh(26)	Foot deformity, MF	Sable
Gourie‐Devi et al. (1984)	India	10	*M*(10)	17−36y	1−10y	Leg(1), thigh(1), leg–thigh(8)	Pain, MF	Stable
Riggs et al. (1984)	USA	1	*M*(1)	34y	8y	Leg(1)	None	NA
Virmani and Mohan (1985)	India	11	*M*(8), *F*(3)	10−46y	0.5−12y	Leg(5), thigh(3)	None	Stable
Visser et al. (1988)	Netherlands	3	*M*(2), *F*(1)	13−25y	4−7y	Thigh(2), leg(1)	None	Stable
Uncini et al. (1992)	Italy	3	*M*(3)	26−42y	4−8y	Leg(1), leg–thigh(2)	Pes cavus	Progressive (1)
Kim et al. (1994)	Korea	11	*M*(8), *F*(3)	17−36y	NA	Lower limbs	Numbness, CP, MF	Stable
Muzio et al. (1994)	Italy	6	*M*(5), *F*(1)	20−51y	6−40y	Leg(2), thigh(2), leg–thigh(2)	Pes cavus	Stable
Kay et al. (1994)	Hong Kong	11	*M*(9), *F*(2)	31−63y	1−28y	Lower limbs	NA	Progressive (7)^a^
Guglielmo et al. (1996)	Italy	5	*M*(6)	50−61y	7−41y	Lower limbs	NA	NA
Saha et al. (1997)	India	10	NA	15−40y	NA	Lower limbs	NA	NA
Hamano et al. (1999)	Japan	2	*M*(2)	33−48y	5−9y	Leg(1), leg–thigh(1)	Pes cavus	Stable
Freitas and Nascimento (2000)	Brazil	16	*M*(8), *F*(8)	4−31y	1−4y	Leg(9), thigh(1), leg–thigh(6)	None	Stable
Dimachkie et al. (2000)	USA	2	*F*(2)	38−48y	6.5−7y	Leg(2)	None	Stable
Castro‐Costa et al. (2000)	Brazil	1	*M*(1)	52y	1y	Lower limb	NA	
Münchau and Rosenkranz (2000)	Germany	1	*M*(1)	45	20y	Leg(1)	None	Stable
Felice et al. (2003)	USA	8	*M*(8)	37−88y	1−5y	Leg(8)	None	Stable
Khandelwal et al. (2006)	India	5	*M*(5)	24y	3.6y	Leg(5)	MF	NA
Weiss,(2005)	USA	1	*M*(1)	58y	10y	Leg(1)	None	Stable
Carvalho and Swash (2007)	UK	10	*M*(8), *F*(2)	32−60y	6−12y	Leg(6), thigh(2), leg–thigh(2)	None	Progressive (3)
Moglia et al. (2011)	Italy	1	*M*(1)	56y	0.5y	Leg–thigh(1)	Cramps, MF	Progressive
Guennoc et al. (2013)	France	1	*M*(1)	46y	15y	Leg(1)	None	Stable
Nalini et al. (2014)	India	55^b^	*M*(51), *F*(4)	9−33y	1−20y	Leg(8), thigh(6), leg–thigh(41)	Cramps	Progressive (1)
Vibha et al. (2015)	India	18	NA	23.5y	1−20y	Lower limbs	NA	Stable
Hui et al. (2018)	China	1	*M*(1)	51y	20y	Thigh(1)	None	Progressive

Abbreviation: *M*(*n*), male case number; *F*(*n*), female case number; NA, not available; MF, muscle fasciculation; CP, cold paresis.

^a^ illness duration less than 5 years.

^b^ the case number may include some reported cases, but it cannot be distinguished.

Neurogenic pattern of EMG, spontaneous activity, and abnormal H reflex indicated a disorder of spinal anterior horn cell in our patients. It was also supported by intact sensations and relatively normal nerve conduction studies in all cases. Chronic neurogenic changes in EMG were confined to the atrophic muscles of the affected limb, except for three patients who simultaneously showed mild abnormalities in the opposite limbs. The phenomenon of contralateral involvement in EMG evidence of chronic denervation was reported in a small number of BMALL patients, which usually was not a progressive indicator for the benign monomelic amyotrophy (Freitas & Nascimento, 2000; Moglia et al., 2011). Intriguingly, no electrophysiological abnormalities were observed in the patients with type IV, which might suggest that this type of congenital‐onset patients would be stable in the disease course without any risks of ALS.

In the current study, we provided the largest number of observations with muscle MRI in BMALL patients. The main features showed different extent of fat infiltration in the involved muscles, but no muscle edema was observed in all patients, which suggested that the lesions were in chronic and stable processes. According to the different distribution of involved muscle groups, we divided the pattern of muscle atrophy into four types. More than half of the patients had fat infiltration in the posterior calf muscles, which had lower frequency than that in Indian and Western patients (Dimachkie et al., 2000; Hamano et al., 1999). MRI muscle examination is very useful to detect the involved muscles, especially deep muscles, and help us locate the affected segments of spinal cord. For example, MRI showed the selective involvement of adductor magnus and tibialis anterior muscles in the patient 10 (Figure 1, type III); the adductor magnus was innervated by a branch of sciatic nerve from L4 to L5, and the anterior tibialis was innervated by peroneal nerve from L4‐S1; therefore, the neuronal lesion should be at anterior sciatic nerve root from L4 to L5 or anterior horn of L4 toL5 spinal cord (Uncini et al., 1992). More intriguingly, MRI showed a well‐proportionate atrophy of both thigh and leg muscles without fat infiltration in the seven patients with type IV, which indicated that the atrophy process might be due to a congenital development disorder.

The muscle pathology varied from almost normal to advanced neurogenic pattern in our patients. The large grouping and muscle fiber hypertrophy suggested that the original lesion might be located at spinal anterior horn cells. The myopathic‐like changes seen in some of the sections may be secondary to occur in ALS and various neurogenic atrophies (Chen et al., 2018). Therefore, the significance of muscle biopsy was limited to patients with BMALL, because the detailed electrophysiological examination and conventional muscle MRI can offer enough diagnostic evidences for BMALL. It was a limitation not to obtain the muscle biopsy from the patients with type IV, which might be helpful to explore the underlying pathogenesis.

BMALL is a rare condition characterized by wasting of a single lower limb with a stable course. Though labeled as “benign” because of a nonprogressive course, a few deteriorating cases were also reported in BMALL patients (Carvalho & Swash, 2007). Similarly, two patients developed to ALS at the 39th month and 56th month after initial evaluation in our follow‐up. However, it was difficult to determine whether these patients with BMALL could progress to ALS at a late stage because about 10% of the patients with ALS still appeared as pure motor neuron involvement in the lower limb 6 years after the disease onset (Berg‐Vos et al., 2003). Interestingly, we found that patients with ALS progression were all in the type II group, though the statistical analysis showed a borderline but negative significance between different groups. Future study with a large number of cases might elucidate the definite relation between clinical outcome and classification of subgroup.

The cause of BMALL is still uncertain, though monomelic amyotrophy of the lower limb has a definite neurogenic origin. BMALL has several similitudines with Hirayama disease (HD). Both syndromes are common in the Asian countries, asymmetrically involve a single limb (lower limb in BMALL and upper limb in HD), and have an insidious onset and a self‐limiting course. Therefore, the BMALL might have a similar pathogenesis with HD that is wildly reported in more cases and intensively investigated in the pathogenic aspects (Foster et al., 2015).

This study had some limitations that need to be explicitly acknowledged. First, it was a retrospective study; thus, some clinical data were incomplete that would lower the clinical significance, for example, the panel of ganglioside antibodies and CSF examinations were not conducted in all patients; therefore, it was difficult to exclude the possibility of immune‐related motor neuropathy in some patients. Similarly, the post‐polio syndrome could not completely be excluded in some old patients, though no history of poliomyelitis and negative IgM antibody refuted the condition (Abrar & Ahmad, 2015). Second, the EMG results were retrospectively collected in these patients, so the examined muscles were not normalized in each patient, which undermined the reliability of diagnostic protocols. Additionally, EMG data were collected from 3 different hospitals, the normal values of the electrophysiological test were a little different in different clinics, so the examined values were just expressed as medians and quartile interval, and not emphasized the absolute values and change range. Third, the follow‐up time was not enough to differentiate BMALL from ALS in some patients. Commonly, a follow‐up > 3–4 years was considered a useful criterion to exclude the majority of patients with ALS (Berg‐Vos et al., 2003; Weiss, 2005). Although 28 of 37 patients had more than five‐year follow‐up plus disease duration, it was reasonable to follow‐up BMALL patients as long as possible.

In summary, it was conclusive that the monomelic amyotrophy of the lower limb had a neurogenic origin, though it was a syndrome comprising a group of conditions with different etiology. Muscle MRI was useful to exactly localize the distribution of involved muscles. The muscle atrophy can roughly be divided into four types on the basis of the distribution of fat infiltration on MRI. Patients with type II showed a tendency toward ALS, but patients with type IV maintained a clinical stabilization without any risks of ALS.

## CONFLICT OF INTEREST

The authors declare that they have no competing interests.

## AUTHOR CONTRIBUTION

WL and XH contributed to analysis, interpretation, and drafting. CS, WH, ZY, CR, and WH contributed to the acquisition and analysis of data. LJ and JK performed the pathological study. ZM and ZMH performed the electrophysiological analysis. YS and HD contributed the study design and drafting the manuscript. HD contributed funding acquisition.

### PEER REVIEW

The peer review history for this article is available at https://publons.com/publon/10.1002/brb3.2073.

## Supporting information

Table S1‐S3

## Data Availability

All relevant data are within the paper and its Supporting Information files.
