# Supplementary material for: Benign monomelic amyotrophy of lower limb in a cohort of chinese patients
Source: Brain Behav. 2021 Mar 2;11(4):e02073. doi: 10.1002/brb3.2073 (PMC8035448; doi:10.1002/brb3.2073)
Supplement: Supplementary file 1 — Table S1‐S3 [file BRB3-11-e02073-s001.pdf]

Supplemental data

Benign monomelic amyotrophy of lower limb in a cohort of Chinese patients

Lulu Wang[1*], Han Wen[1], Shuyun Chen[1], Huan Wang[1], Yilei Zheng[1], Ran Chen[1], Jingjing Li[1], Kaiyan Jiang[1], Haijie Xiang[1], Min Zhu[1], Meihong Zhou[1], Sheng Yao[2], Daojun Hong[1,3]

1. Department of Neurology, the First Affiliated Hospital of Nanchang University, Nanchang, China
2. Department of Neurology, the Sixth Medical Center of General PLA Hospital, Beijing, China
3. Department of Neurology, Peking University People Hospital, Beijing, China

Corresponding authors:

Dr. Sheng Yao and Daojun Hong

E-mail: bjyaosheng@sina.com and hongdaojun@hotmail.com

Department of Neurology, The First Affiliated Hospital of Nanchang University

Yong Wai Zheng Street 17#, Nanchang, 330006, P.R.China

Telephone: 86-791-8869-2511; Fax: 86-791-8869-2511

Running title: BMALL in Chinese patients

Table S1. Electromyographic pattern of wasting lower limbs in the BMALL patients.

Case	Spontaneous activity			Motor unit potential			Interference pattern		
	VM	TA	GA	VM	TA	GA	VM	TA	GA
**Type I**									
1	+	-	-	increase	increase	ND	reduced	normal	ND
2	-	-	-	increase	increase	ND	reduced	reduced	ND
3	-	-	-	increase	ND	increase	reduced	ND	reduced
4	-	-	-	increase	normal	ND	reduced	normal	ND
5	-	-	-	increase	ND	normal	reduced	ND	normal
6	-	-	-	increase	increase	ND	reduced	reduced	ND
**Type II**									
7	-	-	-	increase	increase	increase	discrete	discrete	discrete
8	-	+	-	normal	increase	increase	normal	reduced	reduced
9	-	-	-	normal	increase	increase	normal	reduced	reduced
10	-	-	-	normal	increase	increase	normal	discrete	reduced
11	-	-	-	increase	increase	increase	discrete	reduced	discrete
12	-	-	-	normal	increase	increase	normal	discrete	discrete
13	+	-	+	increase	increase	increase	reduced	reduced	reduced
14	-	-	-	increase	increase	increase	reduced	reduced	reduced
15	-	-	-	normal	increase	increase	normal	reduced	reduced
16	-	-	-	increase	increase	increase	reduced	discrete	reduced
17	-	-	-	normal	increase	increase	normal	mixed	reduced
18	-	-	-	normal	increase	increase	normal	discrete	discrete
19	-	-	-	normal	increase	increase	normal	reduced	discrete
20	-	+	-	increase	increase	increase	discrete	discrete	reduced
**Type III**									
21	+	-	-	increase	increase	increase	reduced	discrete	discrete
22	-	-	-	increase	increase	increase	reduced	discrete	reduced
23	-	-	-	normal	increase	normal	normal	discrete	normal
24	-	+	-	increase	increase	increase	reduced	reduced	reduced
25	-	-	-	increase	ND	increase	discrete	ND	discrete
26	-	-	-	increase	increase	ND	discrete	discrete	ND
27	-	-	+	increase	increase	increase	reduced	reduced	discrete
28	-	-	-	normal	increase	normal	normal	reduced	normal
29	-	-	-	increase	increase	increase	reduced	reduced	reduced
30	-	-	-	increase	increase	normal	reduced	discrete	normal
**Type IV**									
31	-	-	-	normal	normal	normal	normal	normal	normal
32	-	-	-	normal	normal	normal	normal	normal	normal
33	-	-	-	normal	normal	normal	normal	normal	normal
34	-	-	-	normal	normal	normal	normal	normal	normal
35	-	-	-	normal	normal	normal	normal	normal	normal
36	-	-	-	normal	normal	normal	normal	normal	normal

37	-	-	-	normal	normal	normal	normal	normal	normal

Abbreviation: VM, vastus medialis; TA, tibialis anterior; GA, gastrocnemius; ND, not done

Table S2. The fat infiltration scores of thigh muscles in the BMALL.

Patient	Quadriceps femoris	Adductor magnus	Adductor longus	Semimembranosus	Semitendinosus	Biceps femoris long head	Biceps femoris brevis	Sartorius	Gracilis
P1	4	0	0	0	0	0	0	0	2
P2	3	1	0	0	0	0	0	0	0
P3	4	0	0	0	0	0	0	0	0
P4	2	1	1	1	0	0	0	0	0
P5	2	3	2	3	2	3	2	2	1
P6	0	1	2	4	4	4	1	0	0
P7	0	0	0	0	0	0	0	0	0
P8	0	0	0	0	0	0	0	0	0
P9	0	0	0	0	0	0	0	0	0
P10	0	0	0	0	0	0	0	0	0
P11	0	0	0	0	0	0	0	0	0
P12	0	0	0	0	0	0	0	0	0
P13	0	0	0	0	0	0	0	0	0
P14	0	0	0	0	0	0	0	0	0
P15	0	0	0	0	0	0	0	0	0
P16	0	0	0	0	0	0	0	0	0
P17	0	0	0	0	0	0	0	0	0
P18	0	0	0	0	0	0	0	0	0
P19	0	0	0	0	0	0	0	0	0
P20	0	0	0	0	0	0	0	0	0
P21	0	1	0	3	3	3	1	0	0
P22	0	4	1	2	0	1	0	0	0
P23	0	1	1	1	0	1	0	0	0
P24	0	3	1	3	0	0	0	0	0
P25	0	1	0	2	1	0	0	0	1
P26	0	4	1	3	3	3	1	0	0
P27	0	4	1	2	0	1	0	0	0
P28	0	2	0	0	0	0	0	0	0
P29	2	1	1	0	0	0	0	0	0

P30	5	3	1	0	0	0	0	2	0
P31	0	0	0	0	0	0	0	0	0
P32	0	0	0	0	0	0	0	0	0
P33	0	0	0	0	0	0	0	0	0
P34	0	0	0	0	0	0	0	0	0
P35	0	0	0	0	0	0	0	0	0
P36	0	0	0	0	0	0	0	0	0
P37	0	0	0	0	0	0	0	0	0

Table S3. The fat infiltration scores of leg muscles in the BMALL.

Patient	Tibialis anterior	Tibialis posterior	Peroneus longus and brevis	Flexor digitorum longus	Extensor hallucis longus	Soleus	Gastrocnemius
P1	0	0	0	0	0	0	0
P2	0	0	0	0	0	0	0
P3	0	0	0	0	0	0	0
P4	0	0	0	0	0	0	0
P5	0	0	0	0	0	0	0
P6	0	0	0	0	0	0	0
P7	1	0	2	2	4	3	2
P8	2	1	1	1	0	1	1
P9	1	3	2	1	1	5	3
P10	2	5	3	4	5	4	3
P11	4	1	1	1	0	2	0
P12	3	2	1	1	1	2	1
P13	0	0	0	0	0	4	3
P14	0	1	0	0	0	5	4
P15	0	0	1	1	1	2	0
P16	0	0	0	0	0	2	1
P17	0	1	1	1	1	3	3
P18	0	2	0	0	0	4	5

P19	0	0	0	0	0	3	4
P20	3	1	0	0	1	0	0
P21	5	1	0	0	1	0	0
P22	2	0	0	0	0	0	0
P23	4	0	1	0	1	0	1
P24	4	0	1	1	0	0	0
P25	0	0	0	0	0	3	3
P26	0	0	1	1	1	2	4
P27	0	0	1	0	0	3	2
P28	0	0	0	0	0	4	4
P29	0	0	0	0	0	2	2
P30	0	0	0	1	2	5	4
P31	0	0	0	0	0	0	0
P32	0	0	0	0	0	0	0
P33	0	0	0	0	0	0	0
P34	0	0	0	0	0	0	0
P35	0	0	0	0	0	0	0
P36	0	0	0	0	0	0	0
P37	0	0	0	0	0	0	0
